# Comparative Study of Healthy Older and Younger Adults Shows They Have the Same Skin Concentration of Vitamin D_3_ Precursor, 7-Dehydrocholesterol, and Similar Response to UVR

**DOI:** 10.3390/nu16081147

**Published:** 2024-04-12

**Authors:** Oktawia Borecka, John J. Dutton, Jonathan C. Y. Tang, William D. Fraser, Ann R. Webb, Lesley E. Rhodes

**Affiliations:** 1 Department of Earth and Environmental Sciences, Faculty of Science and Engineering, University of Manchester, Manchester M13 9PL, UK; oktawia.v@gmail.com (O.B.); ann.webb@manchester.ac.uk (A.R.W.); 2 Division of Musculoskeletal and Dermatological Sciences, School of Biological Sciences, Faculty of Biology Medicine and Health, University of Manchester, Manchester M13 9PL, UK; 3 Photobiology Unit, Dermatology Research Centre, Salford Royal Hospital, Northern Care Alliance NHS Foundation Trust, Manchester Academic Health Science Centre, Greater Manchester M6 8HD, UK; 4 Bioanalytical Facility, Norwich Medical School, University of East Anglia, Norwich Research Park, Norwich NR4 7TJ, UK; jjdutton1@hotmail.co.uk (J.J.D.); w.fraser@uea.ac.uk (W.D.F.); 5 Departments of Clinical Biochemistry and Endocrinology, Norfolk and Norwich University Hospital, Norwich NR4 7UY, UK

**Keywords:** skin, 7-dehydrocholesterol, vitamin D, photobiology, ultraviolet radiation, elderly

## Abstract

Vitamin D_3_ synthesis in human skin is initiated by solar ultraviolet radiation (UVR) exposure of precursor 7-dehydrocholesterol (7DHC), but influence of age on the early stage of vitamin D_3_ metabolism is uncertain. We performed a prospective standardised study in healthy ambulant adults aged ≥65 and ≤40 years examining (1) if baseline skin 7DHC concentration differs between younger and older adults and (2) the impact of older age on serum vitamin D_3_ response to solar simulated UVR. Eleven younger (18–40 years) and 10 older (65–89 years) adults, phototype I–III, received low-dose UVR (95% UVA, 5% UVB, 1.3 SED) to ~35% of the body surface area. Biopsies were taken for 7DHC assay from unexposed skin, skin immediately and 24 h post-UVR, and blood sampled at baseline, 24 h and 7 d post-UVR for vitamin D_3_ assay. Samples were analysed by HPLC-MS/MS. Baseline skin 7DHC (mean ± SD) was 0.22 ± 0.07 and 0.25 ± 0.08 µg/mg in younger versus older adults (no significant difference). Baseline serum vitamin D_3_ concentration was 1.5 ± 1.5 and 1.5 ± 1.7 nmol/L in younger versus older adults, respectively, and showed a significant increase in both groups post-UVR (no significant differences between age groups). Thus, skin 7DHC concentration was not a limiting factor for vitamin D_3_ production in older relative to younger adults. This information assists public health guidance on sun exposure/vitamin D nutrition, with particular relevance to the growing populations of healthy ambulant adults ≥65 years.

## 1. Introduction

Vitamin D is established to be essential for musculoskeletal health. However, older adults are prone to a low vitamin D status [1]. As there is a growing population of >65-year-olds living in developed countries, including in the UK, where almost a quarter (24.4%) of the population is aged 60 or above [2,3], it is imperative to better understand the functional aspects of ageing skin. The early stage of vitamin D_3_ metabolism is one such crucial area for translational research.

Skin contains the precursor for vitamin D_3_ (cholecalciferol) synthesis, i.e., 7-dehydrocholesterol (7DHC), with its exposure to solar ultraviolet radiation (UVR) initiating vitamin D_3_ biosynthesis. This is usually the major source of vitamin D in humans, with oral intake usually making a smaller contribution [4]. Although both vitamin D_2_ and vitamin D_3_ can be obtained orally, only vitamin D_3_ is synthesised by the skin. The 7DHC is found in higher concentrations in the upper layer of the skin, i.e., the epidermis, than the lower layer, i.e., the dermis, and is present in both keratinocytes and fibroblasts [5,6]. It is rapidly photoconverted to pre-vitamin D_3,_ with peak production in the ultraviolet B (UVB) waveband at 295 nm [7]. Subsequently, pre-vitamin D_3_ is converted more slowly to vitamin D_3_ through heat-induced isomerisation. The vitamin D_3_ diffuses through the dermal capillary beds and enters the body’s general circulation [8]; hence, it is the metabolite that links the skin and blood pathways of vitamin D_3_ supply and metabolism. It then undergoes hepatic hydroxylation to 25-hydroxyvitamin D_3_ (25(OH)D_3_), and following this, renal hydroxylation to the active hormone, i.e., 1,25-dihydroxyvitamin D_3_ (1,25(OH)_2_D_3_). Ex vivo studies indicate that less than 15% of 7DHC is converted to pre-vitamin D_3_ by a single low UVR exposure [9]. However, higher levels of UVR exposure, and exposure to the ultraviolet A (UVA) component of solar UVR, can, on the other hand, lead to the production of inactive photoisomers, and if prolonged, might degrade newly formed vitamin D_3_ [9].

A study published in the 1980s involving skin specimens from surgical patients aged 8–92 years reported a negative correlation (r = −0.89) between patient age and epidermal 7DHC concentration, and concluded that a lower vitamin D status in older people could be explained by a lower skin 7DHC concentration [10]. However, the study had some limitations, including the use of skin samples taken opportunistically from different body sites following a variety of surgical procedures; these ranged from leg amputation to reduction surgery and correction of protuberant ears in children [10]. In contrast, a further study using skin from surgical samples and dermatology outpatients (from unspecified body sites) reported no correlation between patient age and skin 7DHC concentration [11]. These earlier studies used liquid chromatography (LC)–UV detection methodology, and no further research addressing skin 7DHC concentration and ageing has to our knowledge been published.

Here, we have performed a standardised comparative study in prospectively recruited groups of younger and older healthy ambulant adults, with the objective firstly to compare the baseline skin content of 7DHC between these age groups, and secondly to examine their 7DHC–vitamin D_3_ response to UVR exposure. Multiple sampling was performed of matched skin sites (for 7DHC) and blood (primarily for vitamin D_3_) pre and post low, sub-erythemal (sub-sunburn) dose of solar simulated UVR (SSR; emission comprising 5% UVB and 95% UVA) in vivo. This replicates the dose and UVB: UVA balance that can be obtained from ambient solar UVR exposure in everyday life. A modern HPLC–MS/MS assay was employed for analyte measurements, including our recently developed methodology for measurement of 7DHC [12,13].

## 2. Materials & Methods

### 2.1. Volunteers

This study was performed in volunteers residing in Greater Manchester, UK, in the Photobiology Unit, Dermatology Research Centre, University of Manchester, based at Salford Royal Hospital, Greater Manchester, UK, in 2019. Inclusion criteria were healthy volunteers, ambulant males or females, of phototype I–III (white Caucasian), and aged 18–40 or 65–89 years. Exclusion criteria were history of skin cancer or photosensitivity, use of a sunbed or sunbathing within the past 3 months, and taking photoactive or bone active therapies, vitamin D > 200 IU (5 μg)/day, or anticoagulants (including aspirin, clopidogrel, warfarin, and propranolol).

Volunteers were recruited through advertisement and the Photobiology Unit database. Four potential volunteers were excluded: three younger adults did not fit the phototype criteria, and one older adult was undergoing immune-suppressant therapy. The North West Greater Manchester Research Ethics Committee gave approval (reference 18/NW/0493) on 25 July 2018, and volunteers completed the study during January–March 2019. The study adhered to Declaration of Helsinki principles, and volunteers gave written, informed consent. The trial is registered on the ISRCTN website, reference ISRCTN72674753.

### 2.2. Protocol

**Figure 1:**
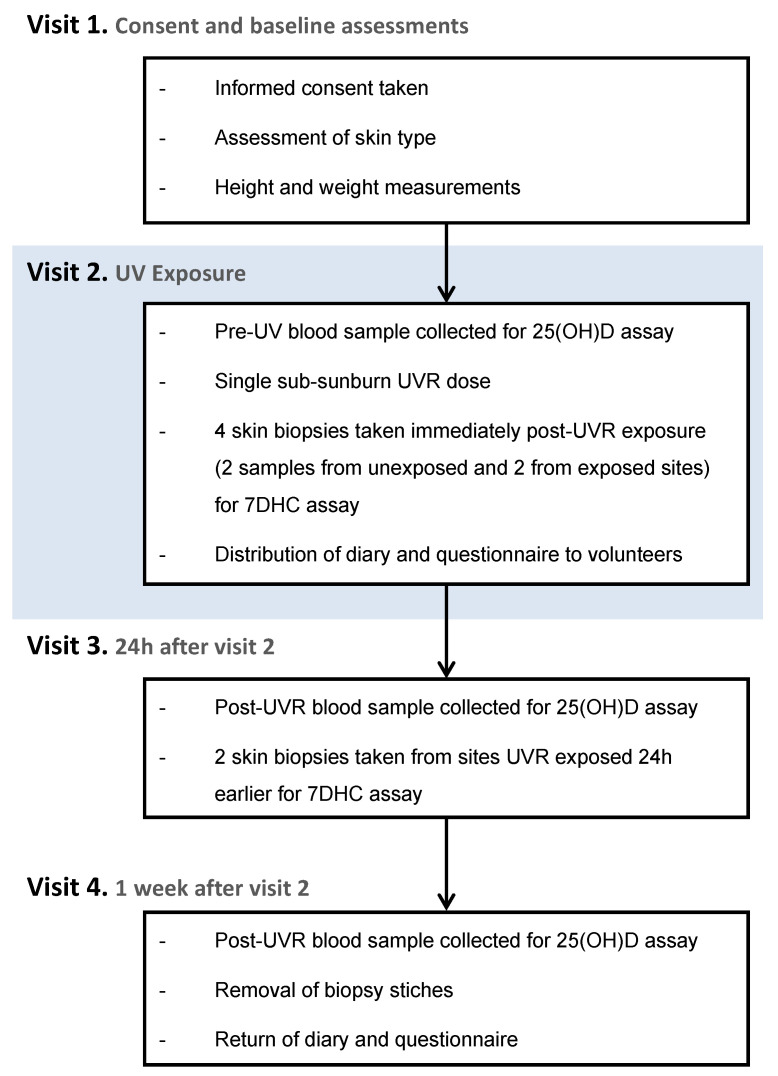
Research protocol flowchart.

The study protocol is shown in a flowchart (Figure 1), with further details of the methodology in the following text.

### 2.3. Phototype Assessment

Volunteers were assessed for sun-reactive skin type (phototype) by researchers based at the Photobiology Unit, Salford Royal Hospital, using a standardised series of questions relating to their history of skin sunburn and tanning responses to sunlight exposure, alongside their physical characteristics, including eye, hair, and skin colour (modified Fitzpatrick system [14]).

### 2.4. Simulated Summer Sunlight Exposures

Volunteers were given a single sub-sunburn SSR dose: 1.3 standard erythemal dose (SED), equivalent to ~15 min UK midday summer exposure. This dose took approximately 6 min 20 s to administer.

A whole-body irradiation cabinet (Philips HB588 Sunstudio, Eindhoven, The Netherlands) was re-fitted with Arimed B fluorescent lamps (Cosmedico GmbH, Stuttgart, Germany), providing UVR emission close to UK ambient midday summer sunlight (290–400 nm; 95% UVA: 320–400 nm, 5% UVB: 290–320 nm). The cabinet emission was characterised with the use of a DTM300 spectroradiometer (Bentham, Reading, UK).

During the UVR exposure, the volunteers lay prone and wore standardised knee-length shorts and a short-sleeved T-shirt, i.e., exposing their hands, forearms, face, and lower legs, equating to ~35% of the body surface area (BSA). A 10 cm × 10 cm area was cut out from one side of their shorts to expose an upper buttock site (UVR biopsy site), and the other buttock was further covered with opaque UVR material (unexposed site). The volunteers wore protective eye goggles.

The study was performed as planned between the months of January and March, in accordance with ambient UVB being insufficient to produce appreciable vitamin D_3_ at the study latitude (Manchester, UK; 53.5° N) during that time period. The UV index (UVI, measure of erythemal effective UV radiation) has been monitored in Manchester since 1997, and daily plots of the UVI can be viewed at https://uk-air.defra.gov.uk/data/uv-index-graphs (accessed on 1 February 2020), where a bell-shaped curve indicates a clear day, and interruptions from such are indicative of clouds. When the UVI is less than 2, it is generally accepted as “safe,” i.e., minimal risk of sunburn and sun protection not required. Vitamin D synthesis is also deemed negligible in any practical scenario of daily life. The UVI only approaches 2 in the middle of the day at the very end of March, and earlier in the year it is far lower.

### 2.5. Skin Biopsies and 7DHC Concentration Analysis

Six 5 mm buttock skin punch biopsies were taken from each volunteer, i.e., two from unexposed skin, two immediately post-UVR (within 30 min of the irradiation), and two at 24 h post-UVR. Extraction, chromatographic separation, and measurement of 7DHC in µg was performed using HPLC–MS/MS, as previously reported [12]. In brief, 7DHC was extracted using ethyl acetate:methanol (1:1, *v*/*v*) and derivatized with 4-phenyl-1,2,4-triazoline-3,5-dione (PTAD) to enhance the ionization efficiency for electrospray ionization mass spectrometry (ESI–MS). Additionally, solid-supported liquid extraction (SLE) was employed to eliminate larger lipids from the 7DHC and reduce potential matrix effects. The LC–MS/MS assay met the validation criteria set by the International Council for Harmonisation. The calibration curve demonstrated linearity, with an average r2 value of 0.997. The coefficients of variation were 11.1% and 4.32% for inter-assay and intra-assay precision, respectively. The lower limit of quantification was 1.6 µg/g, and the upper limit was 100 µg/g. The average recovery of 7DHC through SLE was 91.4%.

Total cellular protein content of the biopsies was used to normalise the 7DHC assay results. This was performed with a colorimetric Lowry-based protein assay using the DC Protein Assay Kit II (Bio-Rad, Hercules, CA, USA). Samples and the calibration curve were prepared following the manufacturer’s protocol [15], and the absorbance was measured using a Multiskan FC plate reader (Thermo Fisher Scientific, Waltham, MA, USA) with an internal shaker at 650 nm. The data obtained from the protein assay were expressed as mg of protein per biopsy, and the 7DHC concentration was expressed as µg of 7DHC per mg of protein.

### 2.6. Blood Sampling, Analysis of Serum Vitamin D Metabolites, and Routine Biochemistry

Blood samples were collected on 3 occasions, i.e., immediately prior to UVR, and 24 h and 7 days post-UVR, and serum was stored at −80 °C prior to analysis. Analysis of serum vitamin D_3_ and vitamin D_2_, 25(OH)D_2_, and 25(OH)D_3_ was performed by LC–MS/MS. Serum 25(OH)D_2_ and 25(OH)D_3_ were measured using a Micromass Quattro Ultima Pt electrospray ionisation mass spectrometer (Waters Corp., Milford, MA, USA), as described previously [13]. The measurement ranges of the assays were 0.1–200 nmol/L for 25(OH)D_2_ and 25(OH)D_3_, calibrated using standard reference material SRM972a from the National Institute of Science and Technology (NIST). The mean coefficient of variation (CV) for intra-assay imprecision across the measuring range of the assays was 4.9% for 25(OH)D_3_ and 8.3% for 25(OH)D_3_, and the cumulative inter-assay CV was ≤7.4% for 25(OH)D_2_ and ≤9.6% for 25(OH)D_3_. Our 25(OH)D assays showed <6% accuracy bias against the Centers for Disease Control and Prevention’s reference method on the Vitamin D External Quality Assessment Scheme (DEQAS). We met the certification performance standards set by DEQAS throughout the time the analyses were performed. Vitamin D_3_ and vitamin D_2_ were analysed using the 4-phenyl-1,2,4-triazoline-3,5h-dione (PTAD) derivatised precursor to product mass ion transitions for vitamin D_3_ (591.4 > 298.1) and vitamin D_2_ (603.5 > 298.1). Serum vitamin D_3_ and D_2_ assays were calibrated using certified pure standards (IsoSciences, King of Prussia, PA, USA) spiked into vitamin D depleted serum (BBSI solutions, Recklinghausen, Germany) and an isotopic-labelled vitamin D_3_-[23,24,25,26,27-13C5] as an internal standard (IsoSciences). The intra- and inter-assay precision coefficients of variation (CV) were 2.2–9.4% across the calibration range of 0–250 nmol/L, with an assay recovery of between 99.7 and 106.3%. The lower limit of quantification (LLoQ) was 1 nmol/L for both vitamin D_3_ and vitamin D_2_; the lower limit of detection (LLoD) was 0.5 nmol/L. All analyses were undertaken by the Good Clinical Laboratory Practice and DEQAS-certified Bioanalytical Facility at the University of East Anglia. Deficient vitamin D status was defined as 25(OH)D < 25 nmoL/L and sufficient status as 50 to 120 nmol/L [16].

Routine biochemistry, including renal function and parathyroid hormone (PTH), was analysed at Salford Royal Hospital, Greater Manchester, UK. Analysis of PTH was with a 2-site sandwich immunoassay using direct chemiluminometric technology (Siemens Centaur XP Intact PTH assay), and routine biochemistry was analysed via the Siemens ADVIA assay.

### 2.7. Sample Size and Statistical Analyses

Sample size was constrained by the number of adults willing to undergo studies involving multiple skin biopsies. The sample size was estimated using confidence interval methodology. Based on data on 7DHC concentration in earlier studies [10,11], a sample size of n = 10 in each age group was estimated to have sufficient power to detect an approximate 2-fold difference in baseline skin 7DHC content between older and younger adults (80% power, alpha = 0.05). With respect to serum vitamin D_3_, using published data [17,18], we estimated n = 10 participants in each age group would give 80% power to detect a mean difference between younger and older groups of approximately 17 nmol/L and 12 nmol/L at 24 h and 7 days post-UVR, respectively, at a 5% significance level.

The 7DHC data were transformed using the y = 1/y equation, after which the data were normally distributed and parametric tests were performed. Vitamin D_3_ results were assessed for normality using the Shapiro–Wilk test and QQ plots (data were normally distributed); similarly, 25(OH)D_3_ data were assessed for normality using the Shapiro–Wilk test and QQ plots. Data were analysed using mixed-effects analysis and the multiple comparisons (Sidak correction) test using GraphPad Prism statistical software (version 8.4.3, 10 June 2020).

## 3. Results

### 3.1. Volunteers

**Figure 2:**
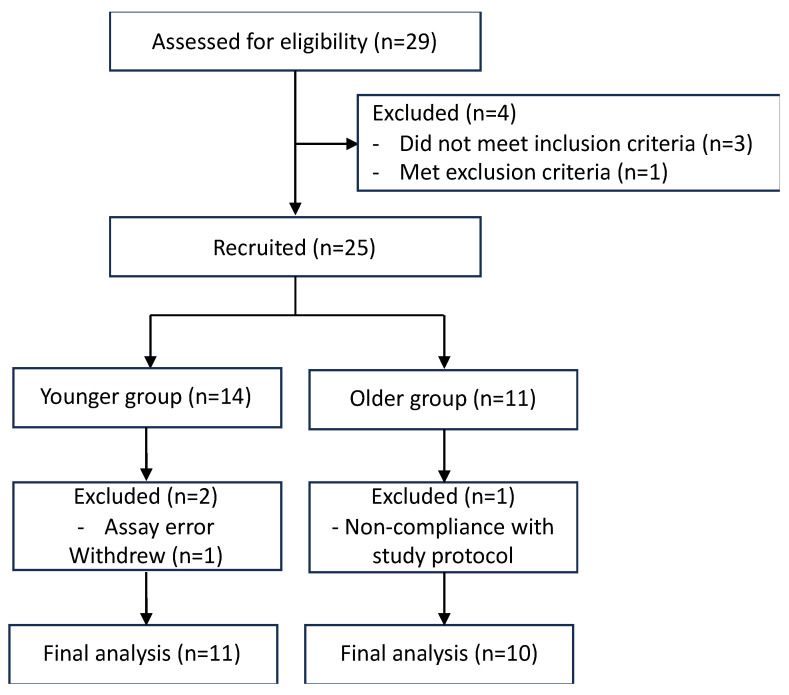
CONSORT patient flow diagram.

Twenty-five volunteers were recruited, with 14 and 11 in younger and older groups, respectively. Post-recruitment, three younger participants were excluded (one withdrew participation, one took a sunny holiday, and one had an abnormally high PTH concentration (13.1 pmol/L); see Figure 2, CONSORT patient flow diagram).

One older volunteer was excluded due to total 25(OH)D being > 2 SD above the mean of the cohort (suggesting the volunteer was taking a higher dose of vitamin D supplements than allowed per study protocol). In total, 11 younger adults and 10 older adults were included in the analysis (Table 1). The 7DHC data were excluded for two younger volunteers in their 24 h post-UVR biopsies due to an error that occurred in the protein assay. One older participant was not able to attend the photobiology unit at 24 h post-UVR, such that no biopsies were collected at this timepoint. Routine biochemistry and PTH results were found to be in the normal range for all the included volunteers.

### 3.2. Skin 7DHC

**Figure 3:**
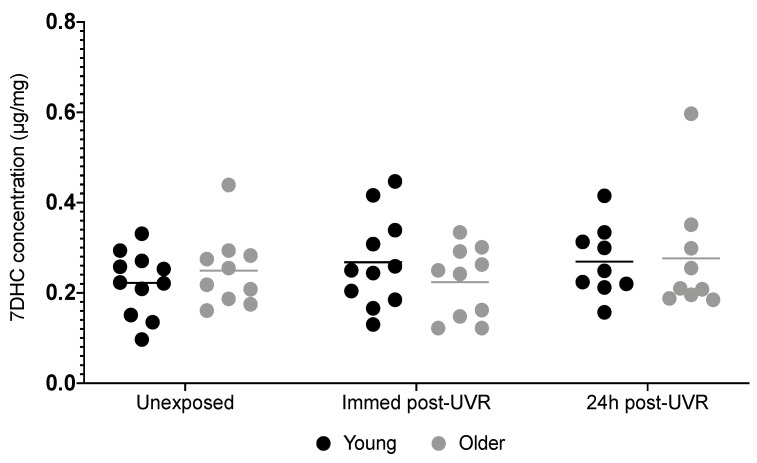
Concentration of skin 7DHC in younger (n = 11/11/9) and older (n = 10/10/9) adults at baseline immediately post-UVR and 24 h post-UVR, respectively. Statistical analyses were performed on transformed data (y = 1/y). Horizontal lines indicate means.

Baseline 7DHC was very similar, and not lower, in the older group versus the younger group (Table 2, Figure 3). No difference was found in 7DHC concentration between younger and older groups at the unexposed/exposed sites in a mixed-effects analysis (*p* = 0.17; F (2,35) = 1.87). The Sidak test-corrected multiple comparisons showed no difference between groups in unexposed (*p* = 0.66), immediately post-UVR (*p* = 0.58), and 24 h post-UVR (*p* = 0.99) skin.

### 3.3. Serum Vitamin D_3_

**Figure 4:**
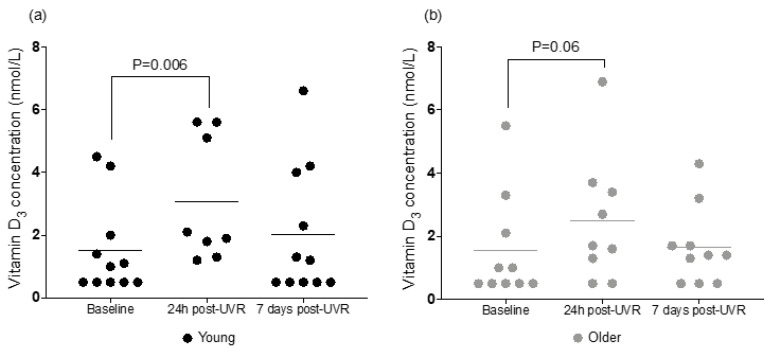
Serum vitamin D_3_ concentration in (**a**) younger (n = 11/8/11) and (**b**) older (n = 10/9/10) adults at baseline, 24 h post-UVR, and 7 days post-UVR. Horizontal lines indicate means.

As the limit of detection of the vitamin D_3_ assay was <1 nmol/L, all values below this level were treated as 0.5 nmol/L for data analysis purposes (Table 2, Figure 4). A mixed-effect analysis showed that time was a significant factor influencing vitamin D_3_ concentrations in both age groups (*p* = 0.002; F (1.645, 27.97) = 8.462), indicating that the single sub-erythemal UVR dose influenced vitamin D_3_ concentration. Age and interaction of time and age were not statistically significant as determinants of vitamin D_3_ level. At baseline, there was no difference in serum vitamin D_3_ between the age groups (the mean in both groups was 1.5 nmol/L). The increase from baseline to 24 h post-UVR was 107% and 67% in younger and older groups, respectively (*p* = 0.006 and *p* = 0.06, respectively, multiple comparisons analysis). In both groups, serum vitamin D_3_ decreased by 7 days, and no differences were found between baseline level and that at 7 days post-UVR.

### 3.4. Serum 25(OH)D_3_

Serum 25(OH)D_3_ and 25(OH)D_2_ were assayed alongside vitamin D_3_ as the standard measure of vitamin D status. We report on 25(OH)D_3_, the only form of 25(OH)D obtained from skin production (Table 2). Mean 25(OH)D_3_ concentrations were not lower in the older group than the younger group, contrary to traditional expectations [1,19], potentially reflecting their health and ambulance. Mixed-effects analysis showed no difference in 25(OH)D_3_ in the groups combined at different timepoints (F (0.1800, 3.240) = 1.416; *p* = 0.16), while multiple comparison testing showed an increase in 25(OH)D_3_ between the baseline and the 24 h post-UVR level (*p* = 0.045). No difference was found in age, time, or interaction of time and age for 25(OH)D_3_ in the younger versus the older groups.

## 4. Discussion

This study was performed to explore whether the cutaneous portion of the vitamin D_3_ biosynthetic pathway differs between younger and older adults. Under standardised conditions, and following our development of an updated skin 7DHC assay [12], we showed that skin 7DHC concentration is not lower in older adults (mean 0.25 µg/mL) than younger adults (0.22 µg/mL), contrasting with a previous study of influence of age on 7DHC [10]. We additionally examined the impact of low-dose SSR in vivo on skin 7DHC and serum vitamin D_3_, i.e., the metabolite bridging the skin and the circulation in the vitamin D biosynthetic pathway. No detectable change occurred in 7DHC concentration of biopsies taken at two timepoints following UVR exposure, potentially reflecting the small percentage of 7DHC converted to pre-vitamin D, while serum vitamin D_3_ increased at 24 h following UVR exposure in both groups. Consistent with this, there was a significant increase in vitamin D status (25(OH)D_3_) from baseline to 24 h following UVR in the combined groups. Our findings indicate that baseline skin 7DHC concentration is not a limiting factor for vitamin D_3_ synthesis in healthy older adults relative to younger adults, and that response to low-dose UVR is similar in older and younger adults.

The data collected in the current study indicated that skin 7DHC concentration was very similar in healthy volunteers in younger and older groups at baseline, and indeed under all conditions examined (unexposed skin, skin immediately post-UVR, and at 24 h post-UVR). This is in contrast with data reported by MacLaughlin and Holick in 1985, where an approximately two-fold lower baseline epidermal 7DHC content was found in surgical patients 70 years of age versus 30 years of age [10]. Their findings could be attributable to the widely differing skin sites sampled between patients and age groups, and also potentially to the suboptimal health status of some tissue [10,16]. Additionally, the skin 7DHC content was normalised using the surface area and wet weight of samples, which can be unreliable due to potential water loss by evaporation; normalisation by dry weight [20] or protein content [21,22], as we have used, is a more consistent method. In a technical study evaluating a skin 7DHC quantification method [11], a seven-fold variation was found in the 7DHC concentration (n = 25, 12.1–80.6 ug/g dry weight), while no correlation was seen with age. Additionally, both of the earlier studies used an LC–UV system to quantify the 7DHC, which can be challenging when compounds of similar molecular weights are found in the extracted mixture.

The amount of 7DHC that is converted to pre-vitamin D_3_ after one UVR exposure appears to be relatively small based on in vitro and ex vivo studies [23]. Thus, it was found that within 5 min of summer sunlight exposure (Boston, MA, USA, 42° N), 3% of 7DHC in methanol in vitro was converted to pre-vitamin D_3_, and after 1 h, ~9% was converted. Inert isomers were formed from pre-vitamin D_3_, which accumulated with time. Similarly, in ex vivo skin experiments, after 3 h of sunlight exposure, only 7% of 7DHC was converted to pre-vitamin D_3_ [23]. In skin specimens irradiated ex vivo with narrowband UVB (295 ± 5 nm; 0.15 J/cm^2^), the conversion rate of 7DHC to pre-vitamin D_3_ ranged from 8 to 17%, with the highest rates in the youngest patients, and at a rate of 24 to 33% if evaluating the epidermis separately; these higher conversion rates may reflect the artificially narrow UV waveband used [10]. MacLaughlin and Holick [10] also irradiated skin specimens from five patients ex vivo with 0.15 J/cm^2^ UVB (295 ± 5 nm) and found that the production of pre-vitamin D from 7DHC in very young people (aged 8 years and 18 years) was two-fold higher than in the three older patients (aged 77 years, 77 years, and 82 years), with mean conversions of 31.9% and 25.8%, respectively. In contrast, our protocol involved UVR exposure of emission spectrum (UVB and UVA) and a dose mimicking ~15 min of midday summer sunlight exposure, and our in vivo approach allowed for the potential influence of blood flow, body temperature, and neighbouring tissues; this improved representation of biology may have contributed to any reduction in 7DHC being too small for detection, and/or the 7DHC may have been rapidly restored. Additionally, the number of post-UVR timepoints that could be subjected to skin biopsy was limited by ethical acceptability.

Changes in circulating vitamin D_3_ following UVR exposure also reflect the early stage of vitamin D metabolism. There was no difference in the mean baseline value of circulating vitamin D_3_ between age groups (both were 1.5 nmol/L), and vitamin D_3_ was observed to increase in both age groups at 24 h after UVR, indicating the conversion of 7DHC to pre-vitamin D_3_. Although vitamin D_3_ increased by 107% and 67% in the younger and older groups, respectively, there was no statistically significant difference between groups. At present, there are no comparable published studies examining the influence of age on circulating vitamin D_3_ response to UVR exposure. However, studies using a variety of protocols have examined the impact of UVR on the level of serum vitamin D_3_, and our baseline measurements are similar to those of other recent studies reporting average levels of ~1.3 and 2.5 nmoL/L [18]. Other studies generally show larger average increases in serum vitamin D_3_ post-UVR [17], which can be accounted for by protocol differences, including higher UVR dose, higher UVB content (converts 7DHC to pre-vitamin D_3_) relative to UVA (in contrast, leads to production of inactive isomers), repeated exposures, and more BSA exposed. Thus, reported average increases in serum vitamin D_3_ post-UVR have ranged from 6.5 nmoL/L in 15 adults aged 23 ± 2.3 years given a single UVR dose of 2.2 ± 0.8 SED to ~50% BSA [18], to 37.7 nmol/L in 10 volunteers aged 48 ± 12 years given 0.66–1.4 SED on 3 consecutive days to ~50% BSA [17]. However, similarly to Libon et al. [18], who measured serum vitamin D_3_ concentration 5 days post-UVR, the serum vitamin D_3_ concentration in our study decreased towards baseline at 7 days post-UVR.

The strengths of our study include its prospective design, use of healthy volunteers, standardised procedures and skin sites, mimicking of casual natural sunlight exposure, use of modern assays, and originality of the approach. We recruited volunteers of matched skin type, and skin samples were collected from a body site protected from the sun. Furthermore, the study was conducted during winter months in the UK, when ambient UVB is insufficient to produce appreciable vitamin D_3_ in skin. The UV irradiation protocol was designed to mimic natural sunlight exposure in humans, using UVR comprising 95% and 5% UVB (similar to ambient UVR in summer), approximately 35% BSA exposure (as when wearing summer clothes, such as T-shirt and shorts), and a low sub-erythemal UVR dose that is believed to be the most efficient in vitamin D_3_ synthesis [9]. We previously showed that such low doses of SSR can increase vitamin D status whilst minimising but not eliminating the risk of skin damage in lighter skin types [24,25].

A limitation of the study is the relatively low number of volunteers, as intensive in vivo multiple biopsy studies are challenging to recruit and perform in higher numbers, and this potentially resulted in missed detection of significant differences. Repeated UVR doses might also be needed to detect a difference in 7DHC concentration following UVR exposure. Similarly, repeated low doses of UVR, as well as a study with a larger volunteer sample size, are indicated to further explore serum vitamin D_3_ differences between age groups post-UVR exposure.

Taken together with inconsistent evidence of the benefits of vitamin D supplements for musculoskeletal health in vitamin D-replete healthy older adults [26], our study may influence assumptions regarding oral vitamin D requirements [27] in the >65-year-old age group. Our findings do not negate the need to be mindful of vitamin D requirements in older adults in residential care or with lower levels of independent mobility, as they can have significantly reduced sunlight exposure and hence lower vitamin D status [28].

## 5. Conclusions

For approximately 40 years, it has been suggested that vitamin D nutrition is compromised in older adults at least partially through lower levels of the skin precursor 7DHC. However, data from our prospective, standardised study indicate that baseline 7DHC concentration is not lower in healthy older adults compared to younger adults. Similar synthesis of vitamin D_3_ was seen in the different age groups upon exposure to a low, sub-sunburn dose of UVR, as can be gained upon casual exposure in daily life. This study provides information assisting health guidance on sun exposure and vitamin D nutrition, which is particularly relevant for the growing populations in many countries of healthy ambulant adults aged >65 years.

## Figures and Tables

**Table 1:** Volunteer demographics *.

	Younger (18–40 Years Old)	Older (65–89 Years Old)
Participants (n)	11	10
Sex (n): male, female	7, 4	6, 4
Age (years)	29.5 ± 6.3	70.2 ± 3.8
BMI [kg/m^2^]	27.1 ± 4.8	26.8 ± 5.6
Skin type I, II, III (n)	3, 2, 6	1, 2, 7

* Mean ± SD values unless otherwise stated. Healthy BMI range is 18.5–25 kg/m^2^.

**Table 2:** Mean (±SD) 7DHC, serum vitamin D_3_, and 25(OH)D_3_ concentrations in combined, younger, and older groups of volunteers.

	Groups Combined	Younger	Older
	7DHC (µg/mg)		
Unexposed	0.24 ± 0.08	0.22 ± 0.07	0.25 ± 0.08
Immediately post-UVR	0.25 ± 0.09	0.27 ± 0.10	0.22 ± 0.08
24 h post-UVR	0.27 ± 0.11	0.27 ± 0.08	0.28 ± 0.13
	Serum Vitamin D_3_ (nmol/L)		
Baseline	1.5 ± 1.5	1.5 ± 1.5	1.5 ± 1.7
24 h post-UVR	2.8 ± 2.0 ^1^	3.1 ± 2.0 ^1^	2.5 ± 2.0 ^1^
7 d post-UVR	1.8 ± 1.7	2.0 ± 2.1	1.7 ± 1.2
	25(OH)D_3_ (nmol/L)		
Baseline	44.2 ± 22.9	37.4 ± 18.9	51.7 ± 25.5
24 h post-UVR	47.7 ± 22.3 ^2^	40.5 ± 18.6	54.1 ± 24.3
7 d post-UVR	46.8 ± 21.0	41.3 ± 19.0	52.7 ± 22.4

^1^ Significant/near-significant value when compared to baseline in this group; *p* = 0.0002, *p* = 0.006, *p* = 0.06. ^2^ Significant value when compared to baseline in this group; *p* = 0.0445.

## Data Availability

Data required to assess the manuscript’s conclusions are provided within the manuscript. Further data relevant to this research can be found on the ISRCTN trial registry website (ISRCTN no. 72674753).
